# Arachidonic acid-derived lipid mediators in multiple sclerosis pathogenesis: fueling or dampening disease progression?

**DOI:** 10.1186/s12974-023-02981-w

**Published:** 2024-01-17

**Authors:** Jelle Y. Broos, Rianne T. M. van der Burgt, Julia Konings, Merel Rijnsburger, Oliver Werz, Helga E. de Vries, Martin Giera, Gijs Kooij

**Affiliations:** 1 Department of Molecular Cell Biology and Immunology, Amsterdam UMC, location Vrije Universiteit Amsterdam, De Boelelaan 1117, Amsterdam, The Netherlands; 2 MS Center Amsterdam, Amsterdam UMC, location VU Medical Center, Amsterdam, The Netherlands; 3 Center for Proteomics and Metabolomics, Leiden University Medical Center, Leiden, The Netherlands; 4 Amsterdam Gastroenterology Endocrinology Metabolism, Amsterdam UMC, Amsterdam, The Netherlands; 5 Amsterdam Neuroscience, Amsterdam UMC, Amsterdam, The Netherlands; 6 Department of Pharmaceutical/Medicinal Chemistry, Institute of Pharmacy, Friedrich Schiller University Jena, Jena, Germany; 7 Amsterdam Institute for Infection and Immunity, Amsterdam UMC, Amsterdam, The Netherlands

**Keywords:** Multiple sclerosis, Arachidonic acid, Prostanoids, Leukotrienes, Inflammation, Demyelination

## Abstract

**Background:**

Multiple sclerosis (MS) is a chronic autoimmune disease of the central nervous system (CNS), characterized by neuroinflammation, demyelination, and neurodegeneration. Considering the increasing prevalence among young adults worldwide and the disabling phenotype of the disease, a deeper understanding of the complexity of the disease pathogenesis is needed to ultimately improve diagnosis and personalize treatment opportunities. Recent findings suggest that bioactive lipid mediators (LM) derived from ω-3/-6 polyunsaturated fatty acids (PUFA), also termed eicosanoids, may contribute to MS pathogenesis. For example, disturbances in LM profiles and especially those derived from the ω-6 PUFA arachidonic acid (AA) have been reported in people with MS (PwMS), where they may contribute to the chronicity of neuroinflammatory processes. Moreover, we have previously shown that certain AA-derived LMs also associated with neurodegenerative processes in PwMS, suggesting that AA-derived LMs are involved in more pathological events than solely neuroinflammation. Yet, to date, a comprehensive overview of the contribution of these LMs to MS-associated pathological processes remains elusive.

**Main body:**

This review summarizes and critically evaluates the current body of literature on the eicosanoid biosynthetic pathway and its contribution to key pathological hallmarks of MS during different disease stages. Various parts of the eicosanoid pathway are highlighted, namely, the prostanoid, leukotriene, and hydroxyeicosatetraenoic acids (HETEs) biochemical routes that include specific enzymes of the cyclooxygenases (COXs) and lipoxygenases (LOX) families. In addition, cellular sources of LMs and their potential target cells based on receptor expression profiles will be discussed in the context of MS. Finally, we propose novel therapeutic approaches based on eicosanoid pathway and/or receptor modulation to ultimately target chronic neuroinflammation, demyelination and neurodegeneration in MS.

**Short conclusion:**

The eicosanoid pathway is intrinsically linked to specific aspects of MS pathogenesis. Therefore, we propose that novel intervention strategies, with the aim of accurately modulating the eicosanoid pathway towards the biosynthesis of beneficial LMs, can potentially contribute to more patient- and MS subtype-specific treatment opportunities to combat MS.

**Graphical Abstract:**

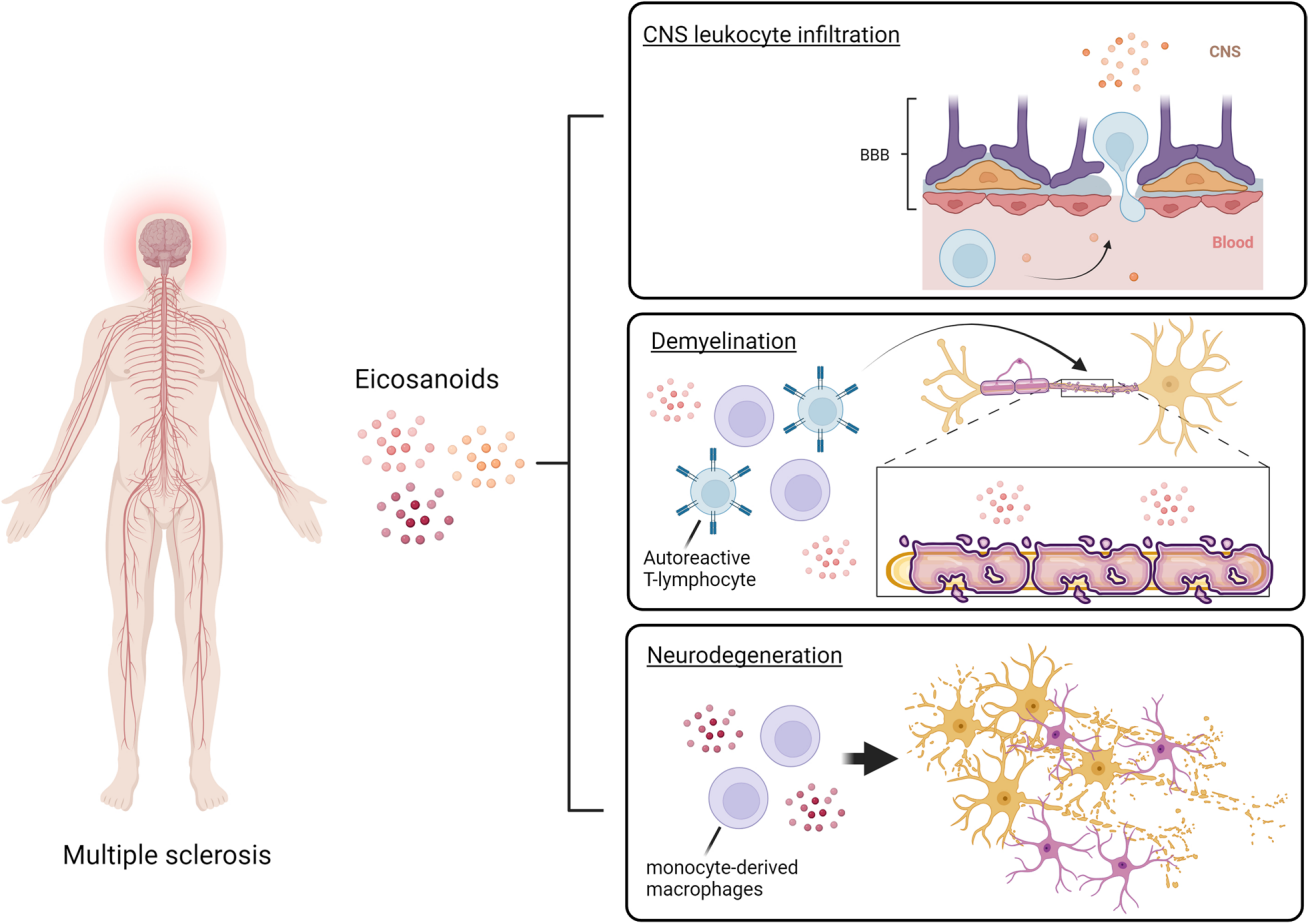

## Introduction

Multiple sclerosis (MS) is a chronic autoimmune disease of the central nervous system (CNS) with an increasing global incidence among young adults (between the age of 20–40 years). In 2020, 35.9 out of 100,000 people were estimated to have MS, which corresponds to 2.8 million people with MS (PwMS) worldwide [1]. Central to the disease is the targeting of the lipid-rich myelin sheath by the immune system, giving rise to its breakdown, a process known as demyelination. As the primary functions of the myelin sheath imply providing nutrients and protection to neurons as well as acting as an electrical insulator for proper neuronal signalling, demyelination often leads to axonal damage and neurodegeneration [2]. In MS, this neurodegeneration can be translated into clinical symptoms, such as vision and cognitive impairments, or physical disabilities (e.g., balance or movement), depending on the location and size of these insults within the CNS [3].

**Fig. 1:**
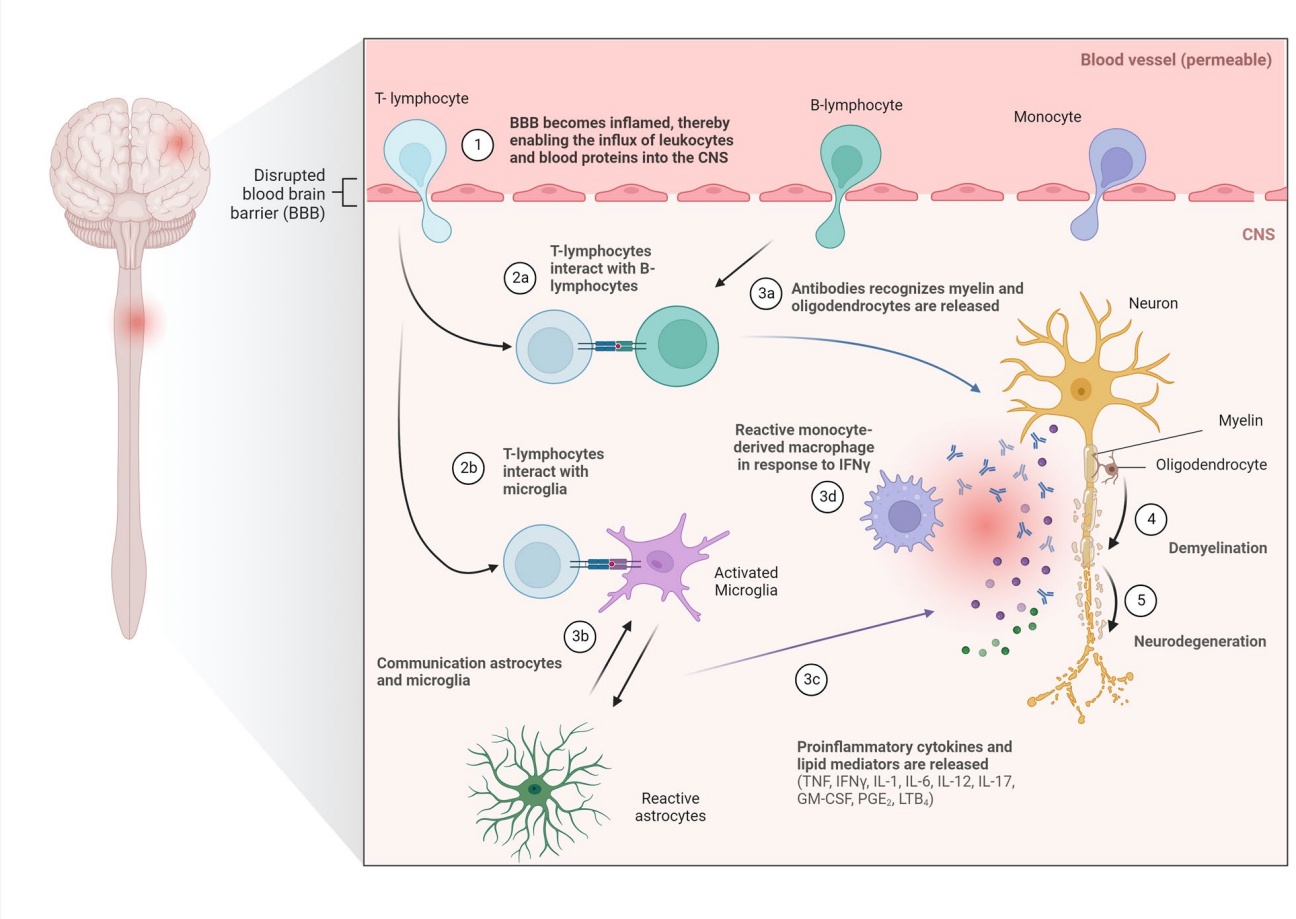
Simplified overview of the traditional perspective on multiple sclerosis pathogenesis. Pathogenesis is mediated by an accumulation and activation of T/B lymphocytes and monocyte-derived macrophages within the CNS (1–2a) and activated microglia and astrocytes (2b, 3b). This leads to the release of a plethora of inflammatory mediators (3a, 3c), targeting myelin sheats and oligodendrocytes surrounding axons (4–5)

Traditionally, MS is believed to start with a primary neuroinflammatory phase characterized by the infiltration of T- and B lymphocytes into the CNS, which, subsequently, attracts peripheral monocytes [3]. Both infiltrating leukocytes as well as locally activated glial cells create a pro-inflammatory environment within the CNS, through the secretion of pro-inflammatory cytokines (e.g., interferon γ (IFNγ), interleukin-1 (IL-1) and tumour necrosis factor (TNF)), neurotoxic factors (reactive oxygen species (ROS)) and matrix metalloproteinases (MMPs) [4]. In turn, this pro-inflammatory environment further triggers glial activation, demyelination and axonal damage [5] (Fig. 1). Moreover, leukocyte infiltration into the CNS is accompanied by a transient disruption of the blood–brain barrier (BBB), a selective barrier comprised of brain endothelial cells, pericytes, and astrocytes that, under healthy conditions, restricts the passage of pathogens, large hydrophilic molecules, and peripheral immune cells into the CNS [6]. BBB disruption and the associated neuroinflammation, besides demyelination and neurodegeneration, therefore, form critical hallmarks of MS pathogenesis, leading to MS lesion formation and disease progression [7, 8]. Nonetheless, large individual differences in the progression of MS among PwMS exist, which can be attributed to numerous genetic and environmental factors [9–13]. For instance, individuals carrying the HLA–DRB1*15:01 allele, individuals who have had a previous infection with the Epstein–Barr virus, and smokers display a higher risk of developing MS. As a result, MS is an extremely heterogeneous and complex disease with an unknown aetiology.

Clinical diagnosis of MS has been roughly divided into three different subtypes: (I) relapsing–remitting MS (RRMS), (II) primary progressive MS (PPMS), and (III) secondary progressive MS (SPMS) [14]. Overall, 85% of PwMS display the RRMS subtype that generally depicts the first phase, defined by recurrent relapses lasting at least a day, followed by partial or full recovery (remission). Here, neuroinflammation, mainly driven by the CNS-infiltrating T- and B-lymphocytes, and demyelination driven by monocyte-derived macrophages are the most common pathological hallmarks. The majority of people with RRMS (PwRRMS), however, gradually develop a more progressive variant of MS, termed SPMS. In this subtype, neurodegeneration becomes more prominent and the innate immune system is suggested to be the main driver of progression (e.g., infiltrating peripheral monocyte-derived macrophages and CNS-resident microglia). Around 15% of PwMS display this progressive course from disease onset and are classified as people with PPMS (PwPPMS). Diagnosis of these subtypes is based on a combination of clinical, biochemical and radiological features, including biomarkers, such as neurofilament light (Nfl), symptom evaluation, and location of demyelinating lesions, as measured by magnetic resonance imaging (MRI). Nonetheless, relapses may hide disease progression during early MS stages and specific disease outcomes, such as progression independent of relapse activity (PIRA), might be more clinically useful instead [15]. Considering the great heterogeneity in disease onset, course, progression and dependence on lesion location, no definitive test for subtype-specific MS diagnosis is currently available.

A better understanding of MS pathogenesis is, therefore, crucial, and current research focuses on biomarker discovery that may enable a more accurate disease course prediction as well as a better distinction between the different MS subtypes to optimize and personalize treatments. Part of this research is focussed on the neuroinflammatory and neurodegenerative components of MS and includes biomarkers, such as Nfl, glial fibrillary acidic protein (GFAP), and IL-1β [16–18]. Recently, bioactive lipid mediators (LMs) derived from ω-3/-6 poly-unsaturated fatty acids (PUFA) have gained interest due to their potential role in MS progression, as they are effective regulators of inflammation, both during onset as well as during inflammation–resolution [19–21]. In addition, derivatives of the ω-6 PUFA arachidonic acid (AA) or eicosanoids [e.g., prostaglandin E_2_ (PGE_2_) and 15-hydroxyeicosatetraenoic acid (15-HETE)], are found to be elevated in PwMS and correlate with clinical parameters, such as the expanded disability status scale (EDSS), Nfl and MRI parameters [19, 20, 22, 23]. These observations suggest that the eicosanoid pathway may fulfil a broader role in MS than solely driving neuroinflammation. This review, therefore, summarizes and discusses the current knowledge on the eicosanoid biosynthetic pathway and its contribution to key pathological hallmarks of MS during different disease stages with a specific focus on AA derivatives in MS pathogenesis.

### The group IVA cytosolic phospholipase A2 (cPLA2-α)-dependent lipid mediator pathway with arachidonic acid (AA) as its substrate

**Fig. 2:**
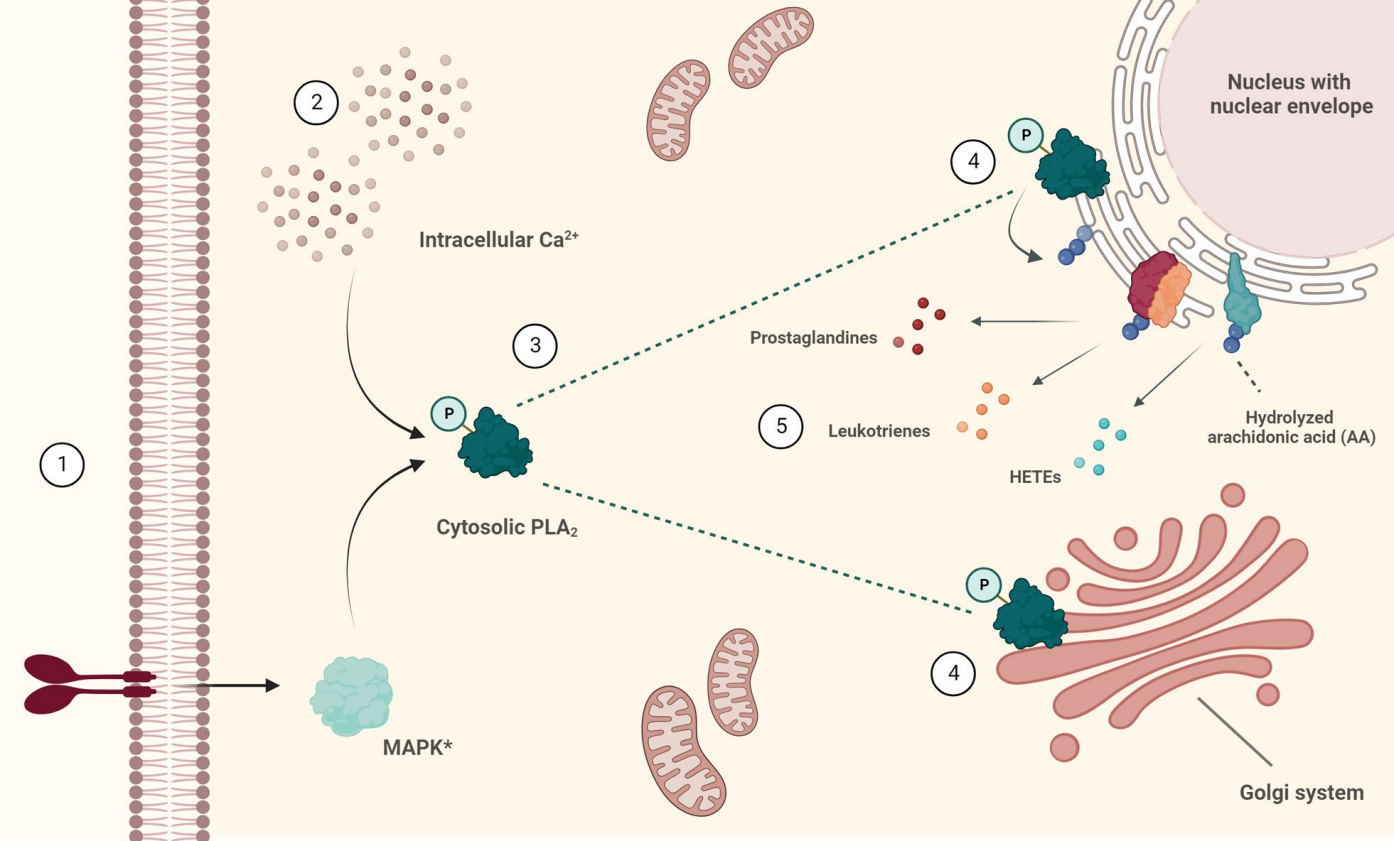
Schematic overview of the molecular signalling that leads to AA hydrolysis from glycerophospholipids in cellular membranes of the Golgi system and the nuclear envelope. Environmental stimuli (e.g., pro-inflammatory cytokines) that either activate the MAPK-signalling pathway (1) or raise intracellular Ca^2+^ levels (2) result in the phosphorylation of group IVA cytosolic phospholipase A2 (cPLA2-α) (3). This results in the translocation of cPLA2-α towards the cellular membranes of the Golgi system and the nuclear envelope, where it interacts with esterified AA incorporated in glycerophospholipids, which will make AA accessible for further metabolism (4). Enzymes with oxidative properties, such as cyclooxygenases (COXs) and lipoxygenases (LOXs), that reside in these cellular membranes can interact with this hydrolyzed AA and convert it into a variety of bioactive LMs (5). *for simplicity, only MAPK without further upstream signalling is shown

AA is an ω-6 PUFA abundantly present in the CNS, liver and muscles, where it is stored in glycerophospholipids within cellular membranes. Upon cellular stimulation, calcium-dependent cPLA2-α is phosphorylated and activated by members of the mitogen-activated protein (MAP) kinase pathway, which promotes its translocation to the Golgi, endoplasmic reticulum (ER) and/or nuclear envelope [24–28]. Here, it catalyses the hydrolysis of AA on the sn-2 position of glycerophospholipids, which triggers the release of AA from the cellular membranes to make it accessible for cytochrome P450 (CYP), cyclooxygenase (COX) and lipoxygenase (LOX) enzymes that can reside at these membranes (Fig. 2) [28, 29]. These enzymes can convert AA further into a plethora of downstream LMs, all having an unique set of biological actions, often defined by interactions with LM-specific receptors (Tables 1, 2).

**Table 1:** Overview of the different prostanoids, their receptors and their described function in MS

	Lipid mediator (LM)	Enzymes required for biosynthesis	Receptors	Described role	References
Thromboxanes	Thromboxane A_2_ (TxA_2_)	TxAS	TP	• Promotes platelet aggregation	[84]
Prostaglandins	Prostaglandin D_2_ (PGD_2_)	Combination of COX-1/2 + H-PGDS or L-PGDS	DP1	• Inhibits the migration and activation of T lymphocytes and basophils	[66, 67]
			DP2	• Promotes T lymphocyte migration	[68]
	15-deoxy-δ (12,14)-PGJ_2_ (15d-PGJ_2_)	Combination of COX-1/2 + H-PGDS or L-PGDS + Non-enzymatically conversion of PGD_2_	PPAR-y	• Suppresses astrocytic and microglial production of pro-inflammatory cytokines TNF, Il-1β • Regulate macrophage migration, proliferation and activation in vitro	[71, 75, 76]
	Prostaglandin E_2_ (PGE_2_)	Combination of COX-1/2 + mPGES-1 or 2	EP1	• Contribute to the disruption of the BBB via matrix metalloproteinase 9 (MMP-9)	[46–48]
			EP2	• Increases in IFNγ and granulocyte–macrophage colony-stimulating factor (GM-CSF) • Induce a pro-inflammatory phenotype in both macrophages and microglia • Increase in COX-2 expression and the induction of apoptosis in rat microglia	[53, 54, 60, 61]
			EP3	–	–
			EP4	• Accumulation of T-helper lymphocytes in the CNS • Differentiation of Th1 lymphocytes • Expansion of Th17 lymphocytes contribute to BBB disruption • Downregulate activation of microglia/macrophage cells	[56–59]
	Prostaglandin F2-alpha (PGF_2α_)	Combination of COX-1/2 + ACR1C1 or ACR1C3	FPα/β	• Indirectly promotes demyelination through glial activation	[70]
	Prostacyclin (PGI_2_)	Combination of COX-1/2 + PTGIS	IP	• Induce Th17 lymphocyte signalling and differentiation in vitro • Prevent pericyte loss and demyelination after LPC treatment • Counteracting the vasoconstrictor and platelet aggregation-promoting role of thromboxane A_2_	[80, 81, 83]

**Table 2:** Overview of ALOX-associated LM and their functions in MS pathogenesis

	Lipid mediator (LM)	Enzymes required for biosynthesis	Receptors	Described role	References
Leukotrienes	Leukotriene B_4_ (LTB_4_)	ALOX5/FLAP complex + LTA_4_ hydrolase	BLT1	• Chemo-attractant for Th17 lymphocytes in vitro	[108, 110]
			BLT2	• Unknown	–
			PPAR-α	• Induces macrophage apoptosis in vitro	[115, 116]
			CysLTR1	• Chemo-attractant for Th17 lymphocytes	[111]
	Leukotriene D_4_ (LTD_4_)	ALOX5/FLAP complex + LTC_4_ hydrolase + Y-glutamyl transferase	CysLTR1/2	• Chemo-attractant for Th17 lymphocytes	[111, 121, 122]
	Leukotriene E_4_ (LTE_4_)	ALOX5/FLAP complex + LTC_4_ hydrolase + Y-glutamyl transferase + LTD_4_ dipeptidase 1/2	CysLTR1/2	–	–
HETEs	5-HETE	ALOX5/FLAP complex	OXER1	• Promote the migration of monocytes	[124–126]
	5-KETE	ALOX5/FLAP complex + 5-HEDH	OXER1	• Promote the migration of monocytes	[124–126]
	11-HETE	Non-enzymatic/ Cytochrome P450/ COX-1/2/ Non-enzymatic	–	• Associates with lipid peroxidation	[125–127]
	12-HETE	ALOX-12	GPR31	• Promote chemotaxis of leukocytes • Induce oxidative stress via an ERK1/2-ALOX-12-ROS pathway • May promote apoptosis signaling of mature OLs	120
			BLT-2	• Promote chemotaxis of leukocytes • Induce oxidative stress via an ERK1/2-ALOX-12-ROS pathway	[134–137]
	15-HETE	ALOX15-1/ ALOX15-B	BLT-2	• Induce foam cell formation	[112, 146, 148–150, 152–154]
			PPAR-y	• Inhibits LTB4-induced chemotaxis of PMN leukocytes in vitro • Promote a pro-resolving phenotype in microglia/macrophages	[146–150, 152–155]

### Cyclooxygenase (COX)-derived prostanoids in MS

The most extensively investigated enzymes of the eicosanoid pathway are COX-1 and COX-2, responsible for the biosynthesis of prostanoids (e.g., thromboxanes, prostaglandins and prostacyclin) through the formation of meta-stable prostaglandin G_2_ (PGG_2_). Where COX-1 is constitutively expressed and thought to have cytoprotective and homeostatic functions, COX-2 expression is tissue-specific, with relatively high expression levels in tissue, such as the kidney, heart and brain, which can be increased in response to growth factors and pro-inflammatory stimuli (e.g., TNF) [30]. Importantly, several studies have shown that COX-2 expression is significantly elevated in PwMS and experimental murine models of MS (i.e., Theiler’s murine encephalomyelitis virus-induced demyelinating disease), specifically in microglia and macrophages [31–34]. These findings have made COX-2 a prominent target in the context of MS pathology, where it is currently considered to mediate both beneficial as well as detrimental processes depending on the biosynthesis of its downstream LMs.

**Fig. 3:**
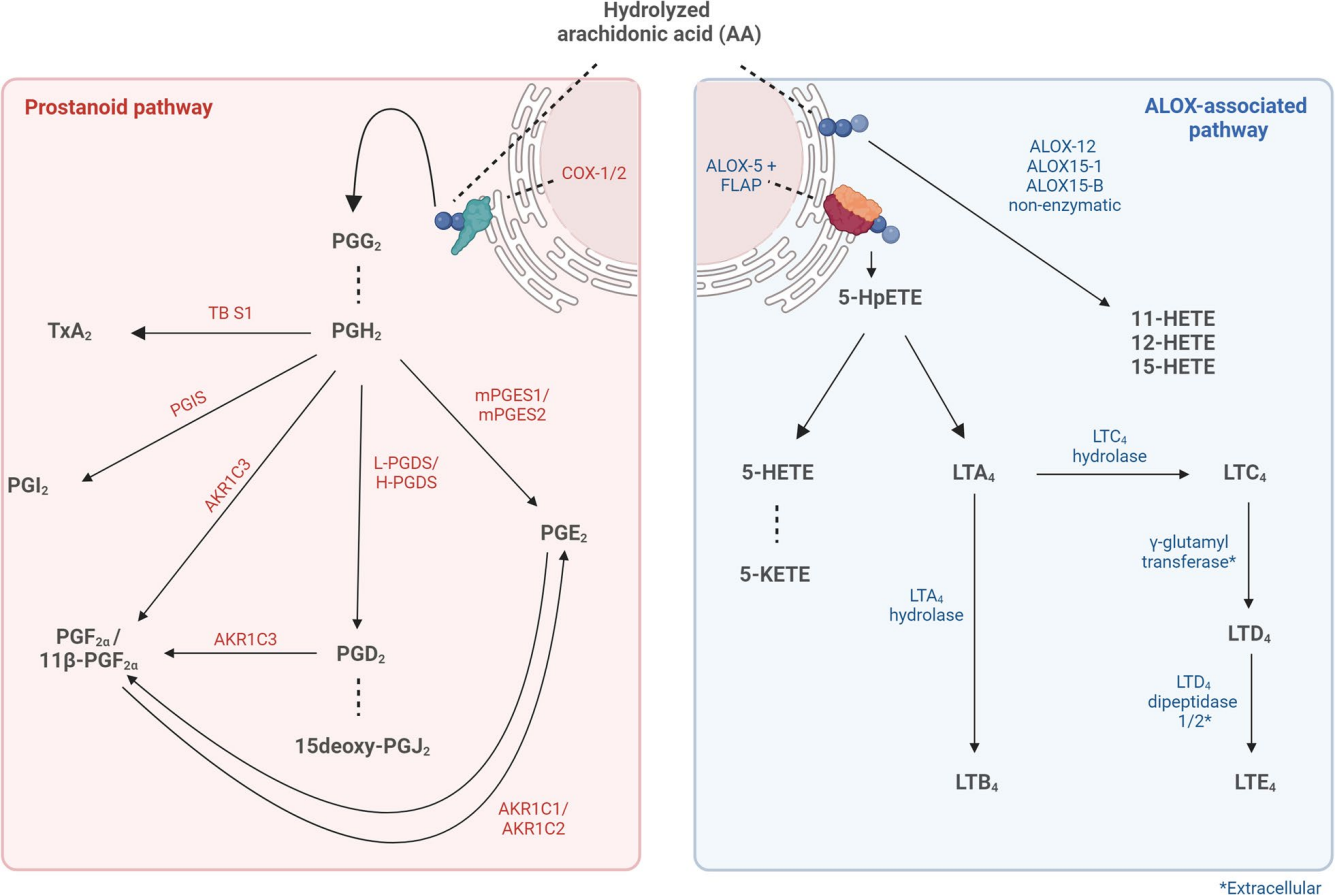
Schematic overview of the variety of COX/LOX associated LMs biosynthesized from the hydrolyzed AA

COX-2 contains two catalytic properties through which it oxidizes the liberated AA, generating the short-living intermediate PGG_2_ (Fig. 3). PGG_2_ is rapidly converted by peroxidase activity into prostaglandin H_2_ (PGH_2_), which forms the central precursor for the synthesis of all other downstream prostanoids, including prostaglandin D_2_ and E_2_ (PGD_2_ and PGE_2_, respectively), prostacyclin (PGI_2_) and thromboxane A2 (TxA_2_). Interestingly, inhibition of COX-2 in the experimental autoimmune encephalitis (EAE) murine model for MS was found to reduce clinical signs by preventing the proliferation of autoreactive T lymphocytes and the production of pro-inflammatory cytokines [35]. However, this approach should be treated with caution, as long-term COX-2 inhibition may translate into severe vascular side effects, such as non-fatal myocardial infarction, non-fatal stroke, or vascular death [36]. In addition, as the synthesis of anti-inflammatory prostanoids, such as 15-deoxy-(12,14)-PGJ_2_ (15d-PGJ_2_), might be affected upon COX-2 inhibition, selective blocking of enzymatic targets further downstream in the prostanoid pathway might be more valuable to combat the neuroinflammatory component of MS to avoid severe side-effects.

#### ***Potential role of PGE***_***2***_***–EP2/EP4 signalling and their dual role during neuroinflammation***

Examples of such downstream enzymatic targets are microsomal prostaglandin E synthase-1 (mPGES-1) and membrane-bound prostaglandin E synthase-2 (mPGES-2), responsible for the biosynthesis of PGE_2_ from PGH_2_ (Fig. 3; Table 1). In MS, PGE_2_ has been linked to the chronicity of neuroinflammation, where several studies have found increased PGE_2_ levels in both serum and CSF of both PwRRMS and people with progressive MS (PwPMS) [20, 23, 37]. Moreover, mPGES-1 was found to be expressed by macrophages in demyelinating lesions, yet a direct link between the increased mPGES-1 expression and PGE_2_ levels has only been shown in EAE mice [38, 39]. Here, the role of mPGES-1-mediated PGE_2_ in disease development seems to be substantial, as mPGES-1 knock-out (KO) mice showed decreased neuroinflammation and demyelination during EAE, that corresponded with lower PGE_2_ levels in their spinal cords [38]. Taken together, these findings suggest that PGE_2_ synthesis is a pivotal contributor to chronic neuroinflammation in MS and that therapeutically targeting of mPGES-1, instead of the more upstream COX-2, may help to attenuate this pathogenic event [40].

Despite these pro-inflammatory characteristics, PGE_2_ should be considered as a versatile LM, depending both on the timing, its concentration and the receptor it binds to. Four receptors [PGE_2_ receptor 1–4 (EP1–EP4)] have been identified to date, through which PGE_2_ can mediate a variety of cellular processes [41] (Table 1). In general, EP1/EP3 receptors promote vasoconstriction and hypertension, whereas EP2/EP4 receptors, in contrast, promote vasodilation and hypotension [42–45]. EP1 and EP3 have been studied in MS or experimental animal models, but are considered to be of little functional relevance and have not been examined in great detail in this context. Nevertheless, EP1 potentially contributes to the disruption of the BBB, as blocking or genetically deleting EP1 in an ischemic murine model led to reduced BBB permeability, presumably through the downregulation of MMP-9, which in MS is found to be elevated in serum of PwRRMS [46–48]. MMPs are enzymes that are involved in BBB breakdown, potentially due to the downregulation of endothelial tight junctions [49]. This suggests that an increase in MMP-9 serum levels facilitates immune cell extravasation into the CNS, potentially in an EP1-dependent manner. Indeed, EP1 may play a larger role in MS development than initially considered, as EP1 gene expression correlates with clinical scores of EAE mice [39]. Notably, PGE_2_–EP3 signalling does not seem to contribute to MS pathology, as MS-related murine models have shown that EP3 is not present in MS lesions, no correlations are found between EP3 mRNA expression and EAE severity and EAE clinical signs are unaffected in EP3 KO EAE mice in vivo [39, 50].

**Fig. 4:**
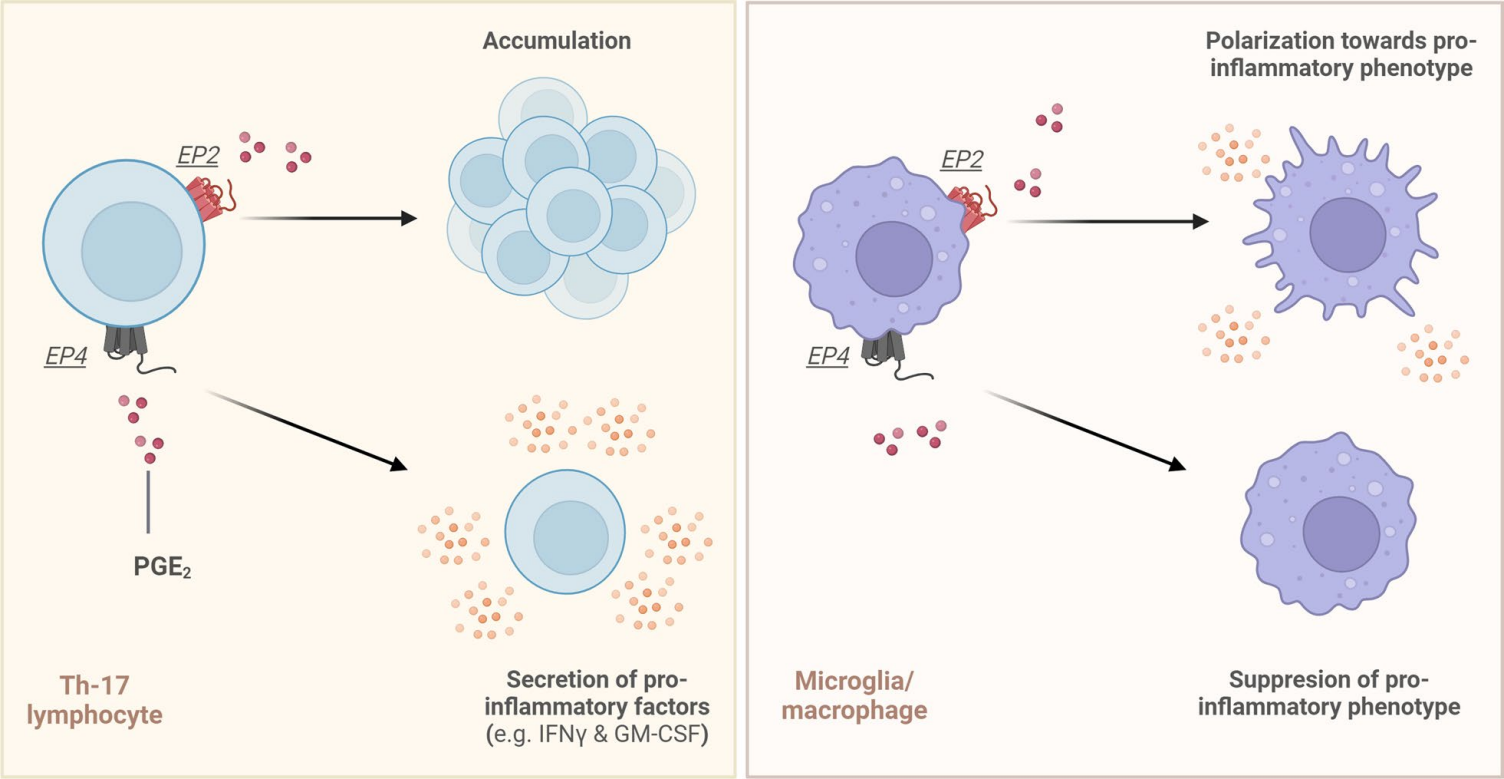
PGE_2_–EP2 and PGE_2_–EP4 signalling in Th17 lymphocytes (left) and microglia/macrophages (right). PGE_2_ can promote the accumulation of Th17 lymphocytes in the CNS by signalling through its EP2 receptor and promotes the secretion of pro-inflammatory factors such as IFN-y and GM-CSF by signalling through its EP4 receptor. However, in monocyte-derived macrophages and microglia EP2 signalling leads to their polarization towards a pro-inflammatory phenotype, while, in contrast, EP4 signalling in these cells results in the suppression of this pro-inflammatory phenotype

In contrast, EP2 and EP4 have been associated with MS pathology as both receptors are involved in the regulation of the adaptive and innate immune system (Fig. 4; Table 1) [41]. Both receptors are, for example, expressed on T-helper lymphocytes as well as on microglia and macrophages, whereas EP2 is also expressed on oligodendrocytes (OLs) [51–53]. Of these immune cells, T-helper lymphocyte type 1 (Th1) and 17 (Th17) are suggested to be the main drivers of MS pathogenesis, as they accumulate in the CNS and actively reinforce a pro-inflammatory environment [54, 55]. Especially EP2 may promote neuroinflammation as its expression is significantly induced on Th17 lymphocytes of untreated PwRRMS as compared to healthy subjects [51]. In turn, treatment of patient-derived Th17 lymphocytes with the EP2-specific agonist butaprost resulted in increased transcription of IFNγ and granulocyte–macrophage colony-stimulating factor (GM-CSF), thus amplifying the inflammatory response, while EP2 overexpression on Th17 lymphocytes of healthy subjects led to similar results [51].

Signalling of PGE_2_ through EP4, on the other hand, is thought to associate with the accumulation of T-helper lymphocytes through increased proliferation in the CNS of PwMS [56]. This is substantiated by decreased numbers of infiltrated CD4^+^ T lymphocytes, monocytes and macrophages in the spinal cord of EAE mice with an EP4 deficiency, where it normally may promote Th1 lymphocyte differentiation and Th17 lymphocyte expansion in an IL-23 and IL-1ß-dependent manner [57, 58]. Furthermore, EP4 signalling may also contribute to BBB disruption, as T lymphocytes of EP4-deficient EAE mice show decreased levels of MMP-9 [59]. Taken together, both EP2 and EP4 likely contribute to T lymphocyte-associated detrimental events during early MS pathogenesis, thereby representing promising therapeutic targets for intervention.

During later stages of MS, the innate immune system and especially CNS-infiltrating monocyte-derived macrophages and CNS-resident microglia may become the main drivers of pathology by creating a chronically inflamed environment in the vicinity of MS lesions. PGE_2_ is also proposed to be involved in this process through EP2/EP4 receptor interactions as it can induce a pro-inflammatory phenotype in both macrophages and microglia through EP2 and, additionally, may promote OLs apoptosis via this signalling pathway [52, 60, 61]. Furthermore, conditional knock-out of EP2 in myeloid cells of lipopolysaccharide (LPS)-challenged mice resulted in reduced hippocampal and cortical IL-6, TNF, IL-1β and inducible nitric oxide synthase (iNOS, a macrophage activation marker) mRNA levels, further demonstrating the pro-inflammatory properties of EP2 signalling [61]. PGE_2_–EP2 signalling led to increased COX-2 expression and induction of apoptosis in primary rat microglia, which could be prevented with an EP2 antagonist [62]. Intriguingly, EP2-deficient EAE mice did not show an attenuation of EAE development, suggesting that the neuroinflammatory role of EP2 signalling in MS is not essential for disease onset or severity [50].

While both EP2 and EP4 signalling leads to an elevation of intracellular cyclic adenosine monophosphate (cAMP) levels, each receptor-dependent signalling cascade showed differential cAMP production profiles, which were also described to be dose-dependent [63]. In addition, the research on the effects of EP4 signalling in macrophages and microglia highlights a more nuanced role compared to the inflammatory role proposed for EP2 (Fig. 4; Table 1). The usage of a selective EP4 agonist on murine microglial cells in vitro attenuated an LPS-mediated pro-inflammatory response and induced transcription of the anti-inflammatory cytokine IL-10 [53]. On the other hand, conditional deletion of EP4 in myeloid cells of mice challenged with LPS led to increased neural COX-2, TNF, IL-6, and IL-1ß expression and elevated F2-isoprostanes levels, a lipid peroxidation marker [64]. This supports the idea that EP4 may have pro-resolving effects in macrophages and microglia, that could occur either by preventing their polarization towards a pro-inflammatory phenotype or skewing it towards a more pro-resolving phenotype, necessary for tissue recovery. A time-dependent factor might be involved, as EP4 expression was found to decrease over time in mouse microglia treated with LPS for 24 h, whereas an inverse effect was seen for EP2 [53]. To this end, cell-type-specific effects of PGE_2_ may take place during the different stages of MS pathology, which may explain why solely silencing EP2 may not yield significant beneficial effects during EAE onset, as EP4 signalling on Th17 lymphocytes can still contribute to the pro-inflammatory CNS environment at this stage. Furthermore, antagonizing the EP4 receptor at different timepoints during EAE development resulted in varying degrees of disease severity [50], further substantiating the complexity and temporal impact of PGE_2_ signalling during the different MS disease phases. Additional insights may be obtained by investigating the effects of an EP4 KO in microglia and macrophages during disease onset as this may hamper EAE development.

#### ***The (anti-)inflammatory or demyelinating potential of PGD***_***2***_*** and its metabolite 15d-PGJ***_***2***_

Besides PGE_2_, other inflammation-mediating prostanoids are identified in PwMS, such as PGD_2_ and its non-enzymatically formed metabolite 15d-PGJ_2_ (Fig. 3). PGD_2_ itself is biosynthesized from PGH_2_ by two distinct synthases; cytosolic hematopoietic PGD synthase (H-PGDS) and the lipocalin-type PGD synthase (L-PGDS) located on the rough ER and nuclear membrane [65]. In plasma of both PwRRMS and people with SPMS (PwSPMS), PGD_2_ levels are found to be elevated [20], where it is proposed to have both anti- and pro-inflammatory properties depending on the G protein-coupled receptor (GPCR) it interacts with: the D prostanoid receptor (DP1) or the chemoattractant receptor–homologous molecule on Th2 cells (CRTH2, also known as DP2) (Table 1). PGD_2_–DP1 signalling is considered to be anti-inflammatory as it inhibits T lymphocyte and basophil migration/activation, whereas PGD_2_–DP2 signalling can promote T lymphocyte migration and thus can be considered pro-inflammatory [66–68]. Intriguingly, PGD_2_ may even play an indirect role in demyelination through the G protein-coupled F prostanoid receptor FP, which will be addressed more extensively in the PGF_2α_ section [69, 70]. Yet, evidence for a direct contribution of PGD_2_ to MS pathogenesis is limited.

The non-enzymatically formed PGD_2_ metabolite 15d-PGJ_2_, however, is known to suppress astrocytic and microglial-mediated production of pro-inflammatory cytokines, such as TNF and IL-1β [71]. It can exert these effects by binding to the nuclear receptor peroxisome proliferator-activated receptor γ (PPAR-γ), which inhibits the inflammation-promoting transcription factors nuclear factor kappa B (NF-κB) and signal transducer and activator of transcription 1 (STAT-1) [72–74]. Next to suppressing pro-inflammatory cytokine production, 15d-PGJ_2_ may regulate macrophage migration, proliferation, and activation in vitro and repress overall EAE development by decreasing toll-like receptor 4 and 9 expression on T lymphocytes in vivo, thereby limiting antigen presentation [75, 76]. However, 15d-PGJ_2_ treatment of undifferentiated mouse oligodendrocyte precursor cells was found to induce apoptosis, suggesting that it may also contain neurotoxic properties [77]. Overall, additional studies are necessary to determine whether PGD_2_ plays a role in MS-associated neuroinflammation and/or demyelination. Nevertheless, its derivative 15d-PGJ_2_ shows several PPAR-γ-mediated anti-inflammatory properties that can be exploited to combat chronic neuroinflammation, although caution is required regarding its potential neurotoxic effects.

#### ***The PGF***_***2α***_*** receptor, FP, mediating demyelination***

Another prostanoid is PGF_2α.,_ that can be biosynthesized either from PGH_2_ or PGD_2_ by the aldo–keto reductase family 1 member C3 (AKR1C3) or from PGE_2_ by the 9-ketoreductase (AKR1C1 and AKR1C2), and exerts its effect through the receptors FP_A_ or FP_B_ (Fig. 3; Table 1) [78]_._ In PwSPMS, peripheral PGF_2α_ levels have been found to be increased, yet, little is known about the specific function of PGF_2α_ in MS [20]. One study showed that a FP antagonist was able to attenuate demyelination of the corpus callosum in the demyelination-inducing cuprizone murine model for MS [70]. Here, a decrease in TNF expression in the corpus callosum was accompanied by a reduction of glial activation and an increase in motor function, suggesting that PGF_2α_–FP signalling enhances glial-mediated demyelination. However, such a direct effect by PGF_2α_ still needs to be addressed and, as briefly mentioned before, this effect of FP signalling could also be mediated by PGD_2_ and PGE_2_, as these prostanoids are also elevated in PwMS and can bind to the FP receptor, albeit with a lower affinity than PGF_2α_ (*K*_i_ = 3.2 nM for PGF_2α_, 6.7 nM for PGD_2_ and 116 nM for PGE_2_ in recombinant HEK293 cells) [69, 79].

#### ***Prostacyclin (PGI***_***2***_***) synthesis and its potential role in neuroinflammation and demyelination***

While it has not been studied extensively in the context of MS, the highly unstable prostacyclin (PGI_2_) has some beneficial, potentially disease-altering properties worth exploring. PGI_2_ is biosynthesized from PGH_2_ by the constitutively expressed enzyme prostaglandin I_2_ synthase (PTGIS, Fig. 3), present in the cytosol of neurons, microglia, and OLs [80]. Once formed, PGI_2_ may exert contrasting, cell-type-specific effects on neuroinflammation or demyelination through the prostacyclin (IP) receptor. For example, stimulating CD4^+^ T lymphocytes with iloprost, a stable PGI_2_-analog, was found to induce an IP-dependent Th17 lymphocyte differentiation and IL-17 production in vitro [81]. In contrast, iloprost treatment was found to prevent pericyte loss induced by lysophosphatidylcholine (LPC) treatment in an in vitro BBB model and diminished LPC-induced demyelination in vivo [82]. In addition, IP-deficiency in EAE mice was found to reduce the infiltration of mononuclear cells into the spinal cord and delayed EAE development, while it did not affect disease severity, suggesting that PGI_2_–IP signalling might be involved in the timing of disease onset but not in overall disease development [83] (Table 1). Finally, PGI_2_ is mostly known to have antithrombotic properties, by counteracting the vasoconstrictor and platelet aggregation-promoting role of thromboxane A_2_ (TxA_2_). This interplay between PGI_2_ and TxA_2_ is essential for a proper cardiovascular homeostasis and should, therefore, be taken into account when considering PGI_2_-associated therapies [84].

#### ***Thromboxane A***_***2***_***, platelet activation and aggregation***

As mentioned above, TxA_2_ is a vasoconstrictor that can promote platelet aggregation [84]. It is biosynthesized from PGH_2_ by the thromboxane-A synthase (TxAS), in a wide variety of cells but especially in platelets, and interacts mainly with the thromboxane prostanoid (TP) receptor (Fig. 3; Table 1). Similar to other prostanoids, TxA_2_ is chemically unstable and degrades quickly through hydrolysis into its inactive, but stable metabolite thromboxane B_2_ (TxB_2_), which is increased in PwMS [20]. Although no conclusive role for TxA_2_ has been defined in MS yet, high platelet activation is seen in PwMS and a direct interaction between platelet aggregation and immunity has been observed consistently [85–88]. In EAE mice, a time-dependent depletion of platelets during disease onset was found to prevent T lymphocyte accumulation in the spinal cord and led to diminished disease and lesion development [87]. More specifically, platelet-activating factors reinforced Th1/Th17 lymphocyte differentiation in early MS and EAE pathogenesis, whereas at later stages of MS, the formation of platelet aggregates and T lymphocytes were associated with diminished T lymphocyte activation [88]. In addition, a low-dose administration of acetylsalicylic acid (ASA, i.e., aspirin), to inhibit platelet activation and aggregation, decreased TxA_2_ and alleviated clinical symptoms of EAE [89]. Still, a direct role of TxA_2_ in these processes in MS remains uncertain, as its instability limits the timeframe for proper detection and the ability to investigate whether TxA_2_ can exert the aforementioned effects in MS pathogenesis before being degraded into TxB_2_.Furthermore, ASA irreversibly acetylates COX enzymes, leading to the complete inactivation of the downstream prostanoid biosynthesis and not solely to that of TxA_2_. Instead, the TP receptor might represent a more interesting target, as isoprostanes, which are free radical-catalysed peroxidation products of AA (e.g., 8-iso-PGF_2_α), are known to promote platelet activation via the TP receptor and have been found to be elevated in the CSF of PwMS as compared to healthy controls [90]. Moreover, TP positively regulates COX-2 expression in endothelial cells and results in increased levels of PGH_2_, thus potentially fuelling the biosynthesis of other prostanoids [91]. Taken together, platelet activation and aggregation may contribute to early MS by reinforcing Th1/Th17 differentiation, although other factors than TxA_2_ might be responsible for this effect via the TP receptor.

To summarize, AA-derived prostanoids encompass several LMs with potent inflammatory or demyelinating properties, which seem to be MS-stage-specific and depend not only on the associated receptor but also on the corresponding cell type. This makes the role of this LM family in MS highly complex, but also provides interesting therapeutic targets for personalized and MS-stage-specific treatment. For example, specific targeting of downstream synthases or receptors, such as mPGES-1 or EP2, might provide more optimal disease-stage-specific therapeutic treatments with high efficacy. However, as most LMs are extremely unstable and versatile in a cell-, receptor- and perhaps even time- and concentration-dependent manner, extensive research is warranted to further understand their exact role in the context of MS pathology.

### Lipoxygenase (LOX)-associated AA-derivatives in MS

Besides the COX-mediated biosynthesis of eicosanoids, an increasing amount of research is focusing on other enzymes with oxygenation properties, such as the lipoxygenases (LOXs), which are thought to be critically involved in microglia-mediated neuroinflammation [92]. A potential reason for this association may involve 5-LOX, which, together with 5-LOX activating protein (FLAP), forms the foundation for the biosynthesis of pro-inflammatory leukotrienes (LTs) (Fig. 3) and is consistently overexpressed in gene expression profiles of peripheral blood mononuclear cells (PBMC)s in PwRRMS [93, 94]. 5-LOX resides in the cytoplasm or nucleoplasm and translocates to the nuclear envelope following stimuli such as stress signals that either increase intracellular calcium levels or promote 5-LOX phosphorylation [95]. At the nuclear envelope, 5-LOX forms an enzymatic complex with FLAP that facilitates the transfer of free AA to 5-LOX. 5-LOX then catalyses the oxygenation of AA, forming 5(S)-HpETE, which, in turn, is rapidly converted into either 5-HETE or into the unstable intermediate leukotriene A_4_ (LTA_4_) by an additional enzymatic cycle. Of these products LTA_4_ is the most interesting in the context of MS as it forms the central precursor for the biosynthesis of other LTs.

Controversy exists regarding the role of 5-LOX in the context of MS. A protective role, for instance, was attributed to 5-LOX during MS pathogenesis based on the observation that EAE progression was exacerbated in 5-LOX-deficient EAE mice [96]. However, in cuprizone mice 5-LOX inhibition with MK-886 attenuated neuroinflammation, motor dysfunction and axonal damage, while it did not reduce the cuprizone-associated demyelination [97]. In addition, administration of flavocoxid, a dual COX-2/5-LOX inhibitor, attenuated EAE pathogenesis presumably by promoting the transition of inflamed microglia towards an anti-inflammatory phenotype [98]. Moreover, in both PwMS and EAE mice, 5-LOX gene expression was upregulated in MS lesions, which was found to be mainly expressed by macrophages in these areas as shown with immunohistochemical analysis [99]. Studies with human monocyte-derived macrophages showed that inhibition of FLAP reduced the biosynthesis of pro-inflammatory LTs, such as leukotriene B_4_ (LTB_4_) [100, 101]. In addition, FLAP inhibition has been applied in in vivo models for several inflammatory diseases, including asthma and atherosclerosis, with some inhibitors successfully being applied in clinical trials, showing the potential of this targeting strategy in the context of MS [102, 103].

A possible explanation for these contrasting effects on both inflammation and EAE disease progression may be attributed to the wide range of metabolites 5-LOX can synthesize, similar to the COX enzymes, as 5-LOX is also involved in the biosynthesis of the pro-resolving lipoxins and resolvins [21]. Nonetheless, in human leukocytes these pro-resolving LMs are present in low quantities and often cannot be detected, in contrast to the abundant release of LTs under inflammatory conditions [104]. Of interest, in contrast to blocking 5-LOX, targeting FLAP in macrophages can efficiently suppress LT formation without reducing resolvin levels [100, 101]. Therapeutic targeting of downstream LT-associated synthases might be a more direct approach to steer the direction of LM biosynthesis towards more beneficial LMs during specific MS disease stages and targeting of the LTB_4_ receptor 1 (BLT1) may provide such a tool.

#### ***LTB***_***4***_***: a potent chemoattractant for migrating leukocytes towards the CNS***

**Fig. 5:**
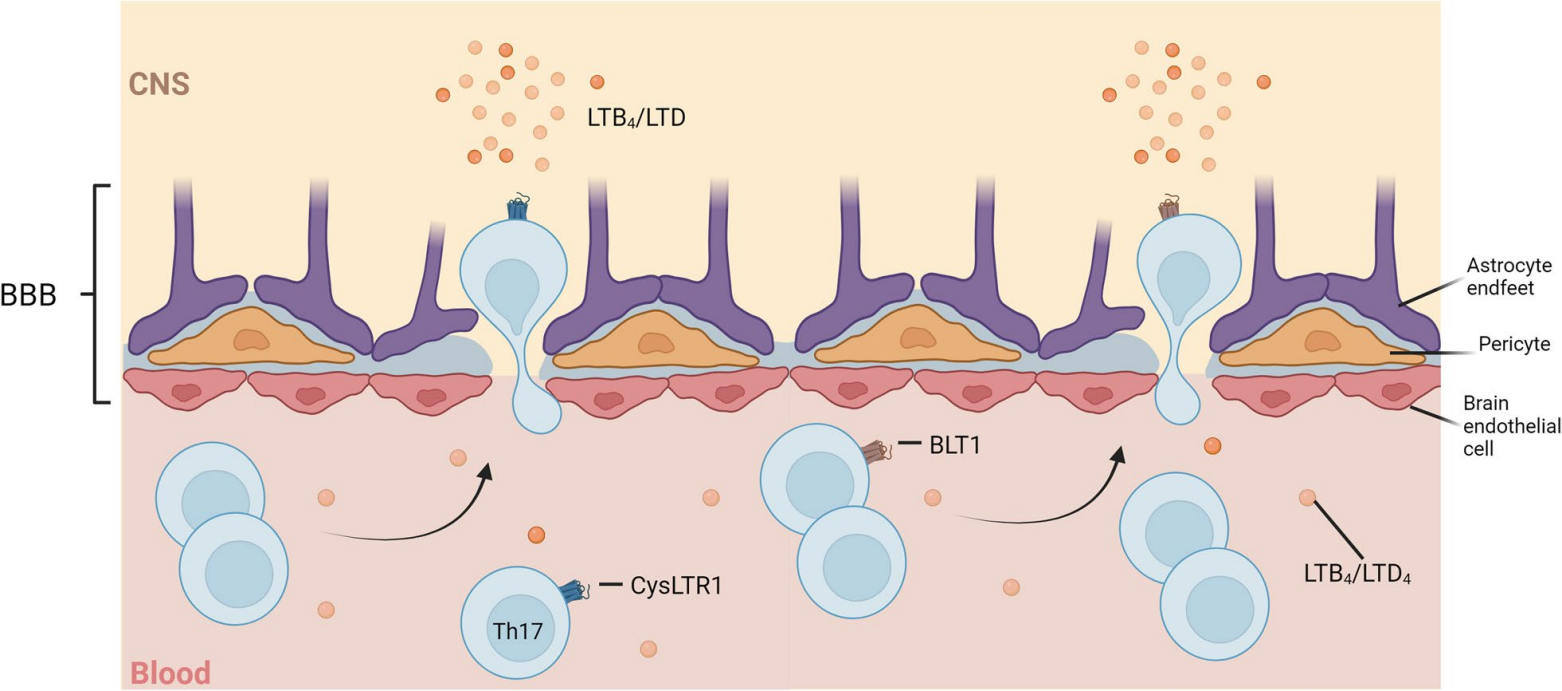
Hypothetically, LTB_4_/LTD_4_ can act as chemo-attractants through either the BLT-1 or CysLTR1 receptor on Th-17 lymphocytes thereby mediating their influx across the disrupted BBB towards high LTB_4_/LTD_4_ levels in the CNS of MS patients

LTB_4_ is the most common leukotriene implicated in MS pathogenesis and is biosynthesized from LTA_4_ by the LTA_4_ hydrolase (LTA_4_H) (Fig. 3). It exerts its effect mainly via two GPCRs called BLT1 and BLT2, and through PPAR-α, through which it promotes chemotaxis of lymphocytes, T lymphocyte activation, and ROS production (Table 2) [105–108]. In MS, these chemoattractant properties can mediate the migration of Th17 lymphocytes into the CNS, as BLT1 is not only highly expressed on Th17 lymphocytes, but these cells also migrate along an LTB_4_-dependent gradient in vitro (Fig. 5) [108]. In addition, LTB_4_ levels are found to be almost twice as high in the CSF of people with clinically active MS when compared to healthy controls [109]. This suggests that the CNS infiltration of lymphocytes during MS depends to a certain extent on signalling through the LTB_4_–BLT1 axis. In line with this, BLT1 deficient mice show reduced CNS infiltration of T lymphocytes, neutrophils and peripheral monocyte-derived macrophages during EAE [110]. This coincided with a delay in EAE onset in combination with reduced disease severity and diminished production of pro-inflammatory cytokines, such as IFNγ, TNF, IL-6 and IL-17, stressing the importance of the LTB_4_–BLT1 axis in EAE pathogenesis. Intriguingly, migration of Th17 lymphocytes towards a high LTB_4_ concentration also diminished after treatment of EAE mice with montelukast, a type 1 cysteinyl leukotriene receptor (CysLTR1)-specific antagonist, indicating that the LTB_4_-associated chemotaxis of Th17 lymphocytes may not solely depend on the BLT1 receptor [111].

LTB_4_–BLT2 signaling, on the other hand, might be less involved in mediating neuroinflammation, as LTB_4_ is known to have a much lower affinity for BLT2 than for BLT1 [112]. In addition, other AA derivatives, such as 12-HHTrE, a byproduct of TxA_2_, and 15-HETE, which will be addressed later in detail, display a greater affinity for BLT2 and compete with LTB_4_ for BLT2 binding, but not BLT1 [112, 113]. As a result, BLT2 may mediate distinct biological as well as pathophysiological processes compared to BLT1, such as epidermal wound healing [114]. Finally, LTB_4_ signaling via PPAR-α could exert anti-inflammatory effects, as signaling via this route was found to play a role in macrophage apoptosis in vitro [115, 116]. However, no clear link with MS pathology can be drawn for LTB_4_–PPAR-α signaling considering that EAE progression and severity in PPAR-α KO EAE mice was similar to that of WT EAE mice, and PPAR-α protein levels, unlike PPAR-γ, are unaltered in the CSF of PwMS [117, 118]. Together, LTB_4_ potentially influences MS pathogenesis via the BLT1 receptor, through which it can promote the chemotaxis of Th17 lymphocytes into the CNS, where they fuel a pro-inflammatory environment. Specific BLT1 blocking or preventing LTB_4_ biosynthesis may, therefore, be considered as potent therapeutic strategies, especially during the early stages of MS pathology, which are dominated by profound lymphocyte CNS infiltration. Finally, while most studies focus on the role of LTB_4_ on lymphocyte CNS infiltration which indirectly leads to demyelination and neurodegeneration, a local, more direct contribution of LTB_4_ to these pathological events is also plausible given its pro-inflammatory nature, as suggested by the autocrine effects of LTB_4_ on microglial activation through the BLT1 receptor [119].

#### *Linking cysteinyl-leukotrienes to lymphocyte infiltration*

Besides LTB_4_, LTA_4_ can also be converted into cysteinyl leukotrienes (CysLTs), which comprises LTC_4_, LTD_4_ and LTE_4_ (Fig. 3) [120]. This LT cascade starts with the conversion of LTA_4_ into LTC_4_ a process that is catalysed by the LTC_4_ synthase in conjugation with glutathione. LTC_4_ is subsequently secreted into the extracellular space via the multi-drug resistance protein 1 (MRP-1), where it can be further converted into LTD_4_ and LTE_4_ by extracellular synthases, such as γ-glutamyl transferase and LTD_4_ dipeptidase-1 and -2 (Fig. 3). All CysLTs exert their actions via one of the two GPCRs CysLTR1 or CysLTR2, where LTD_4_ has a high affinity for CysLTR1 and both LTC_4_ and LTE_4_ for CysLTR2 (Table 2) [120]. Of these LMs, LTD_4_ is considered to be the most relevant for MS due to its high affinity for CysLTR1, the receptor associated with Th17 lymphocyte migration and EAE disease severity as previously described [111]. Selective blocking of CysLTR1 with Montelukast prevented this migration towards high LTB_4_ concentrations, but also towards high LTD_4_ concentrations in vitro [121, 122].

The importance of CysLTR1-signaling in MS is gaining more interest as CysLTR1 is found to be elevated in the blood of PwMS, with an increase on CD4^+^ T lymphocytes, but also on astrocytes and microglia in MS lesions compared to normal appearing white matter in *post-mortem* brain tissue [123]. In addition, more CysLTR1-positive Th-lymphocytes were found inside MS lesions of these PwMS as compared to normal appearing white matter. As elevated levels of both LTB_4_ and LTD_4_ have been observed in the CSF of clinically active PwRRMS, it is tempting to speculate that these LTs might be related to the increased number of Th-17 lymphocytes via CysLTR1 [109, 123]. Nevertheless, whether these findings are directly linked to one another remains to be addressed.

### Hydroxyeicosatetraenoic acids (HETEs) in MS

A generally understudied LM subclass of AA-derivatives in the context of MS are the hydroxyeicosatetraenoic acids (HETEs), of which 5-, 11-, 12- and 15-HETE will be discussed (Fig. 3). In our previous work, we have shown that relative plasma levels of 5-HETE were increased in PwPMS as compared to PwRRMS and healthy controls and correlated positively with EDSS and serum Nfl levels, suggesting a link with disease progression [19]. However, no evidence for local 5-HETE levels in the CNS is available to further substantiate these initial findings. In addition, its oxidised metabolite 5-OxoETE, which is formed under oxidative stress by the microsomal enzyme 5-hydroxyeicosanoid dehydrogenase (5-HEDH), may be of importance in neuroinflammation [124]. 5-OxoETE can function as a chemoattractant for monocytes synergistically with chemokine (C–C motif) ligand (CCL) 2 and 7. It also acts as a potent activator of GM-CSF secretion by monocytes via the oxoeicosanoid receptor 1 (OXER1), also known as the GPR170 in humans (Table 2) [124–126]. Based on these properties, 5-OxoETE may promote monocyte migration towards lesions, where they can induce a pro-inflammatory environment by GM-CSF secretion. However, whether 5-HETE, 5-OxoETE or GPR170 signalling actually contribute to MS disease progression warrants further investigation.

#### *11-HETE, lipid peroxidation and other pathological hallmarks of MS*

One of the AA-metabolites of this subclass is 11-HETE, which can be biosynthesized either by COX-1/2, CYP, or non-enzymatically as byproduct of AA auto-oxidation [127, 128]. Mainly due to this auto-oxidative biosynthesis, 11-HETE is described as a marker for lipid peroxidation, a process known to occur in MS and thought to be related to inflammation, demyelination and neurodegeneration [129, 130]. However, no receptors for 11-HETE have been identified to date and 11-HETE itself has never been linked to MS before. Nevertheless, other lipids associated with lipid peroxidation have been studied in the context of MS, for example, increased levels of the classical oxidative low-density lipoproteins (ox-LDL) and high-density lipoproteins (ox-HDL), for instance, have been found in both the brain, plasma and CSF of PwMS, where their neurotoxic properties are considered to promote oxidative damage [131, 132]. Thus, it remains to be determined whether the AA-metabolite 11-HETE displays these neurotoxic properties as well.

#### *12-HETE: potential promotor of neuroinflammation and ROS-mediated demyelination*

The platelet-type 12-lipoxygenase (12-LOX or ALOX12) is the predominant producer of 12-HETE, and is found to be increased in the plasma of both PwPMS and PwRRMS in remission [20, 133]. In contrast, 12-LOX expression in PBMCs of PwRRMS during a relapse was found to be significantly lower than that of healthy subjects [20]. Despite the lack of a clear contribution of this LM to MS pathology, one can speculate that 12-HETE can have significant pathological implications in inflammatory diseases by promoting the chemotaxis of leukocytes and induction of oxidative stress through receptor interactions (i.e., GPR31 and BLT2) [114, 134–136]. 12-LOX-associated ROS production was, for example, found to induce apoptosis of mature OLs both in vitro and in vivo through an ERK1/2–12-LOX–ROS pathway, suggesting that 12-LOX, and presumably 12-HETE, may contribute to demyelination in MS [137]. Furthermore, 12-HETE can both stimulate and inhibit platelet aggregation thereby affecting T lymphocyte accumulation and differentiation, critical processes during early MS and EAE pathogenesis [87, 138]. Additional studies are, therefore, required to assess the contribution of 12-LOX and 12-HETE to MS pathogenesis with a specific focus on demyelination, mediated by the loss of OLs, and neuroinflammation.

#### *15-HETE: a link between lipids and MS lesions?*

The last LM of this subclass of monohydroxylated AA metabolites addressed here is called 15-HETE and can be biosynthesized from AA by several lipoxygenases, including 15-lipoxygenase-1 (15-LOX-1 or ALOX15) and 15-LOX-2 (or ALOX15B) (Fig. 3). In our recent work, relative plasma levels of 15-HETE, together with disease duration, Nfl and GFAP, were revealed as possible predictors of MS disability (as measured by EDSS) in PwPMS [19]. In addition, negative correlations were observed for 15-HETE with MRI parameters such as total brain and deep grey matter volumes in PwPMS and indicate a potential link between this AA-metabolite and neurodegenerative processes. These findings are in line with other studies, where increased levels of 15-HETE were observed in CSF and plasma of PwMS [20, 23, 37]. Aside from peripheral production, we hypothesize that local 15-HETE is biosynthesized primarily by 15-LOX-2 in demyelinated areas, potentially as a result of hypoxia or oxidative stress, as these stimuli have been linked to induction of 15-LOX-2 gene expression [131, 139–142]. 15-LOX-1, which also generates 15-HETE, may contribute to these elevated 15-HETE levels as well, since efferocytosis (referred to as the effective clearance of apoptotic cells) initiates ALOX15-1 expression in macrophages in vitro and has been observed in demyelinating areas [143, 144]. However, ALOX15-1 moderately converts AA into 15-HETE and may prefer the ω-6 PUFA linoleic acid (LA) as its substrate [145].

The potential function of 15-HETE in the CNS of PwMS is still relatively unknown, although 15-HETE can regulate several cellular processes via its two receptors: BLT2 and PPAR-γ (Table 2) [112, 146, 147]. Detrimental effects might be ascribed to 15-HETE as it is able to induce ROS production, apoptosis and macrophage foam cell formation, processes that are all observed in PwMS [147–150]. The latter process is, in particular, interesting in MS, as CNS-infiltrating macrophages are known to become oversaturated with oxidized lipids derived from the deteriorating myelin sheath, thereby turning into foam cells [151]. 15-HETE may promote this lipid uptake as it can induce membrane glycoprotein CD36 expression in these cells, a scavenger receptor that recognizes oxidized phospholipids and lipoproteins and mediates their internalization [152, 153]. Increased 15-LOX-2 expression is also found in atherosclerotic plaques known to contain foamy macrophages and silencing of ALOX15-B in an atherosclerotic mouse model resulted in decreased lipid accumulation and inflammatory markers in macrophages [150]. On the other hand, 15-HETE was also found to inhibit LTB_4_-induced chemotaxis of polymorphonuclear (PMNs) leukocytes in vitro and may promote a pro-resolving phenotype of macrophages/microglia via binding to the nuclear PPAR-γ receptor, thereby potentially promoting tissue recovery in the MS lesion vicinity [154, 155]. Additional studies, including in vivo studies, are, therefore, crucial to unravel the relevance between the elevated 15-HETE levels in PwPMS and MS-associated neuropathological events.

## Summary and future perspectives

MS is a heterogeneous disease of the CNS, where current therapeutic strategies are mainly focussed on symptom management, predominantly by targeting specific parts of the immune system. Disease-modifying therapies including interferon beta, leukocyte migration inhibitors and monoclonal antibodies that result in lymphocyte depletion are currently on the market, which generally reduce relapse rate, but, unfortunately, have a limited effect on disease progression and are accompanied by unwanted side effects. Therefore, a high and unmet need remains to design therapeutic strategies that incorporate anti-inflammatory, remyelination-promoting and/or neuroprotective effects to slow down disease progression with as little side-effects as possible.

The involvement of AA-derived LMs in various pathogenic processes such as the MS-associated neuroinflammation, demyelination and neurodegeneration suggest that they may have versatile and disease-altering properties. By delving deeper into the role of LMs in MS, one may gain new insights into MS subtype-specific occurrences, ultimately leading to the development of subtype-specific intervention strategies and accompanying biomarkers. In this review, we substantiated the importance of targeting receptors associated with LMs or the downstream biosynthetic enzymes to dampen pathogenic processes and fuel the protective characteristics of LM biosynthesis in MS while minimizing the risk of side effects. This necessity is demonstrated by the example of COX-1/2 inhibitors such as nonsteroidal anti-inflammatory drugs that are used to alleviate flu-related symptoms, highlighting their beneficial and anti-inflammatory properties. However, these medications (i.e., COX-2-selective coxibs) have also been associated with severe cardiovascular-associated side effects, presumably due to imbalances in the prostanoid pathway, for example, between PGI_2_ and TXA_2_ (Fig. 3). COX-2 inhibition affects the metabolism of a broad range of LMs, and each of these could be detrimental or beneficial in the context of MS, such as PGE_2_, PGI_2_, PGD_2_ and its derivative 15d-PGJ_2_. Instead, targeting downstream biosynthetic enzymes in this pathway, such as mPGES-1 and 2, should be considered as improved intervention strategies as these enzymes comprise the final step for PGE_2_ biosynthesis and their inhibition may, therefore, have no negative impact on beneficial prostanoids. Similarly, targeting LTB_4_/LTD_4_ metabolism by modulation of enzymes involved in the leukotriene biosynthetic pathway, such as FLAP, LTA_4_H or LTC_4_H, may also be beneficial in MS as this may potentially disrupt the (LTB_4_-related) chemotaxis signal that drives T lymphocytes infiltration into the CNS of PwMS during early disease stages.

Moreover, cell-type-specific targeting of LM-associated receptors bears the potential to affect disease pathogenesis. For example, blocking the PGE_2_ receptors EP2 and EP4 and the LTB_4_/LTD_4_ receptors BLT-1 and CysLTR1 on Th-lymphocytes during early MS stages (e.g., relapse phase) and EP2 on microglia/macrophages at later stages (e.g., progressive phase) may provide useful tools to influence disease-specific pathological events. Other parts of the AA pathway, such as monohydroxylated HETEs, have not been investigated thoroughly, yet may provide additional targets for intervention. Overall, in this review we provide evidence that the AA metabolome is strongly intertwined with pathological processes in MS and indicate the need for strategies targeting this molecular pathway, to create novel patient- and MS subtype-specific therapeutic options against MS.

## Data Availability

Not applicable.

## Abbreviations

AA: Arachidonic acid
AKR1C: Aldo-keto reductase family 1 member C
ASA: Acetylsalicylic acid
BBB: Blood brain barrier
BLT: Leukotriene B_4_ receptor
COX: Cyclooxygenases
cPLA2-α: Group IVA cytosolic phospholipase A2
CRHTH2: Chemoattractant receptor–homologous molecule on Th2 cells
cAMP: Cyclic adenosine monophosphate
CYP: Cytochrome P450
CysLTR: Cysteinyl leukotriene receptor
DP: D prostanoid receptor
EAE: Experimental autoimmune encephalitis
EDSS: Expanded disability status scale
EP: Prostaglandin E_2_ receptor
ER: Endoplasmic reticulum
FLAP: 5-Lipoxygenase-activating protein
FP: F prostanoid receptors
GM-CSF: Granulocyte–macrophage colony-stimulating factor
GPCR: G protein-coupled receptor
GSH: Glutathion
HETE: Hydroxyeicosatetraenoic acid
H-PGDS: Hematopoietic PGD synthase
HIF-1α: Hypoxia-inducible factor 1 alpha
IFNγ: Interferon gamma
IL: Interleukin
IP: Prostacyclin receptor
LM: Lipid mediator
iNOS: Inducible nitric oxide synthase
LOX: Lipoxygenase
LPC: Lysophosphatidylcholine
L-PGDS: Lipocalin-type PGD synthase
LPS: Lipopolysaccharide
LT: Leukotriene
LTA4H: Leukotriene A_4_ hydrolase
MAP: Mitogen-activated protein
MMP: Matrix metalloproteinase
mPGES-1: Microsomal prostaglandin E synthase-1
mPGES-2: Membrane-bound prostaglandin E synthase-2
MRP-1: Multi-drug resistance protein 1
MS: Multiple sclerosis
NF-κB: Nuclear factor kappa B
Nfl: Neurofilament light
NO: Nitric oxide
OLs: Oligodendrocytes
OXER1: Oxoeicosanoid 1
Ox-HDL: Oxidized high-density lipoprotein
Ox-LDL: Oxidized low-density lipoprotein
PBMC: Peripheral blood mononuclear cells
PG: Prostaglandin
PGHS: Prostaglandin endoperoxide synthase-1
PGI_2_: Prostacyclin
PGIS: Prostaglandin I_2_ synthase
PPAR-γ: Peroxisome proliferator-activated receptor γ
PMN: Polymorphonuclear
PMS: Progressive multiple sclerosis
PPMS: Primary progressive multiple sclerosis
PUFA: Polyunsaturated fatty acid
PwMS: People with multiple sclerosis
PwPMS: People with progressive multiple sclerosis
PwPPMS: People with primary progressive multiple sclerosis
PwRRMS: People with relapsing-remitting multiple sclerosis
PwSPMS: People with secondary progressive multiple sclerosis
ROS: Reactive oxygen species
RRMS: Relapsing–remitting multiple sclerosis
SPMS: Secondary progressive multiple sclerosis
STAT-1: Signal transducer and activator of transcription 1
Th17: T-helper lymphocyte type 17
TNF: Tumor necrosis factor
TP: Thromboxane receptor
Tx: Thromboxane
TxAS: Thromboxane-A synthase
5-HEDH: 5-Hydroxyeicosanoid dehydrogenase
15d-PGJ_2_: 15-Deoxy-(12,14)-PGJ_2_
